# Supplementary figures and images for: Context-dependent ciliary regulation of hedgehog pathway repression in tissue morphogenesis
Source: PLoS Genet. 2023 Nov 9;19(11):e1011028. doi: 10.1371/journal.pgen.1011028 (PMC10662714; doi:10.1371/journal.pgen.1011028)

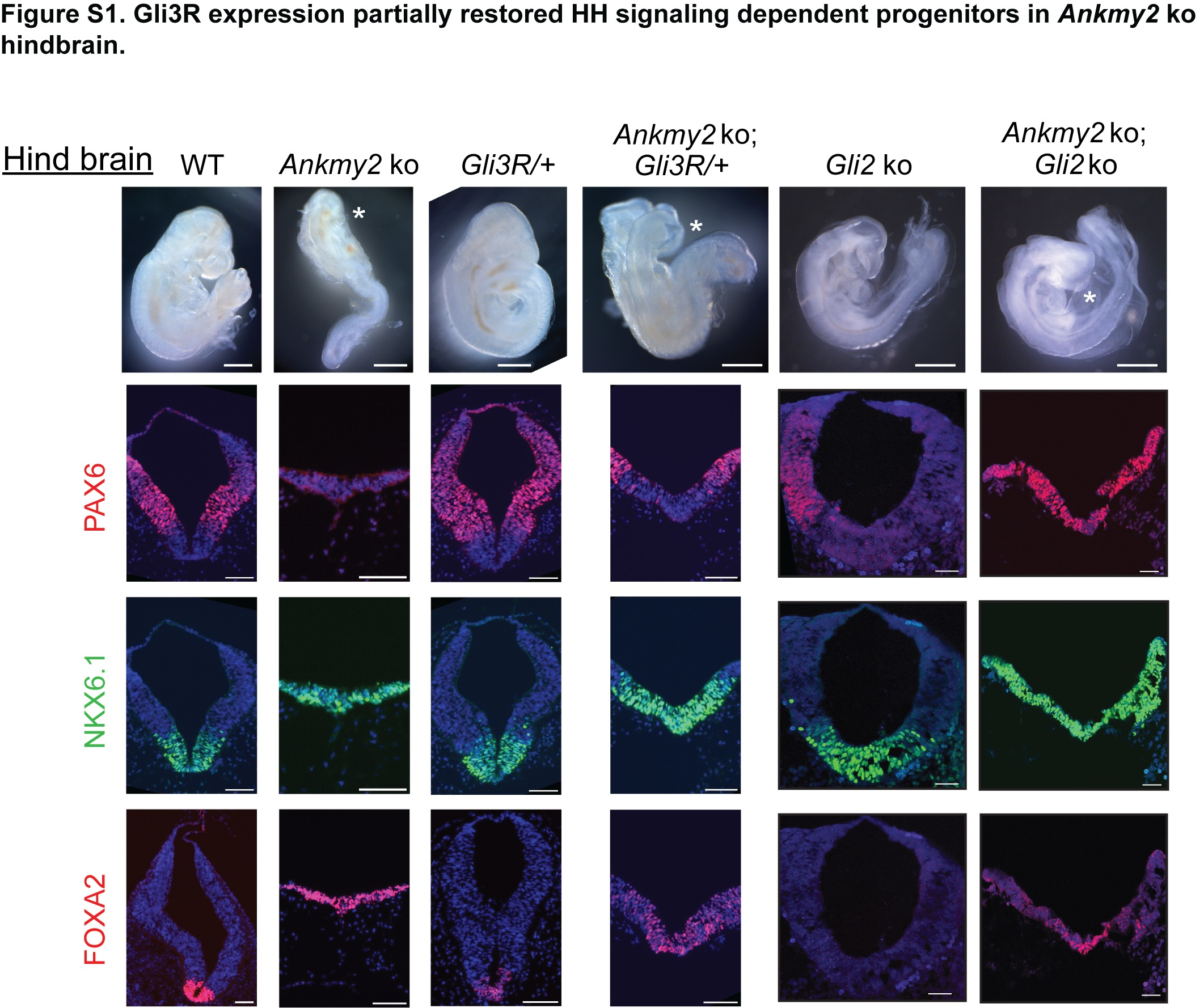

Supplement: S1 Fig — Top, panels show bright-field images of wildtype (wt), Ankmy2 ko, Gli3R/+, Ankmy2 ko; Gli3R/+, Gli2 ko, Ankmy2; Gli2 double ko, and whole-mount embryos at E9.25 of the following genotypes: wild-type (n = 5), Ankmy2 ko (n = 5), Gli3R/+ (n = 4), Ankmy2 ko; Gli3R/+ (n = 4), Gli2 ko (n = 3), Ankmy2 ko; Gli2 ko (n = 3). Exencephaly (marked by asterisk) persists in Ankmy2 ko and double mutants. Scale: 500 μm. Bottom, panels show hindbrain neural tube horizontal sections immunostained using designated markers. All images are counterstained with DAPI. Scale: 100 μm. (TIF) [file pgen.1011028.s001.tif]

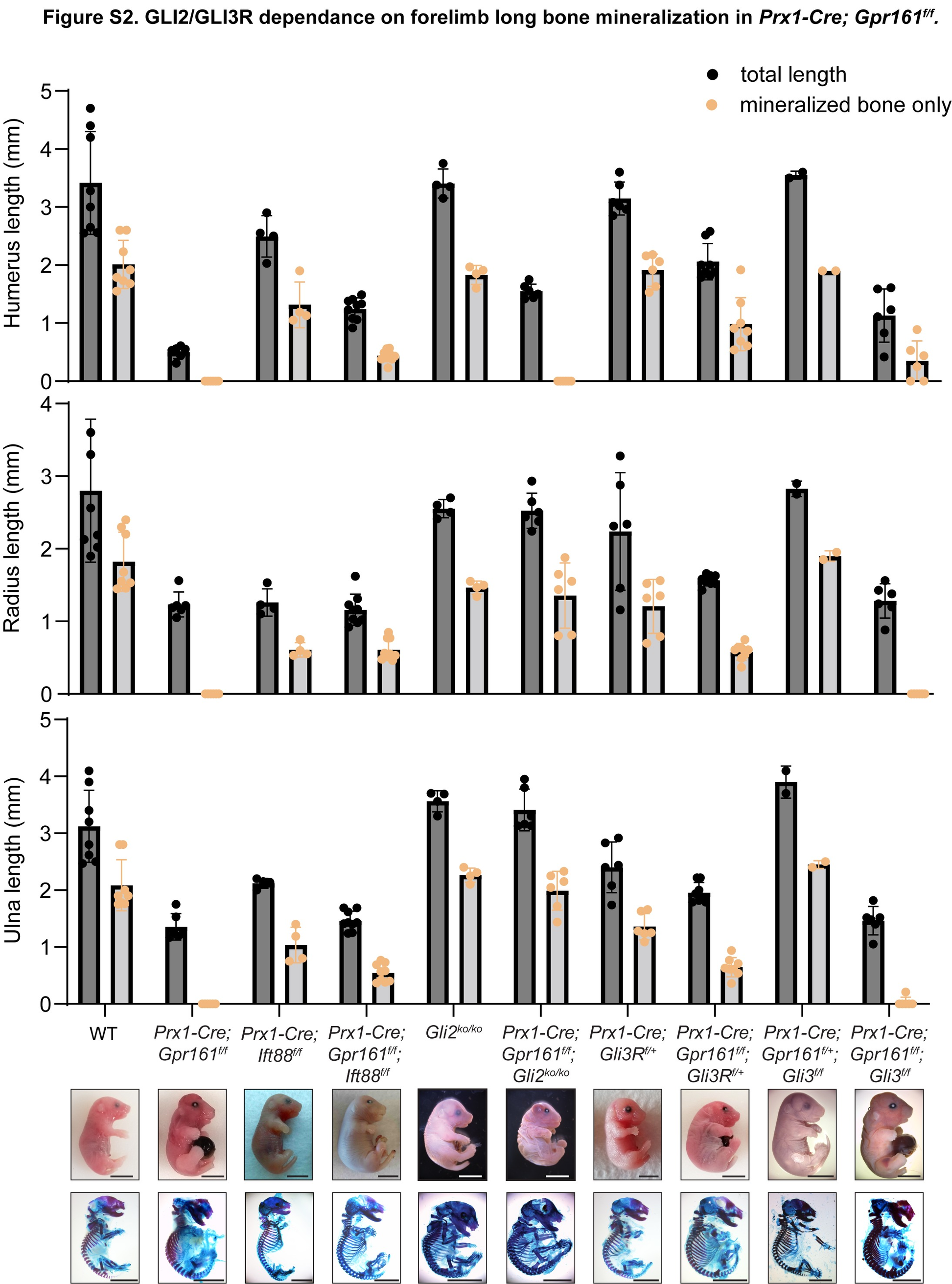

Supplement: S2 Fig — Quantification of forearm long bone primordia lengths in Fig 4 shown. Each data point represents individual bone primordia quantified. Alcian blue (unmineralized cartilage) and alizarin red (mineralized cartilage and bone) staining of E18.5 embryos shown below. Scale, 5 mm. (TIF) [file pgen.1011028.s002.tif]

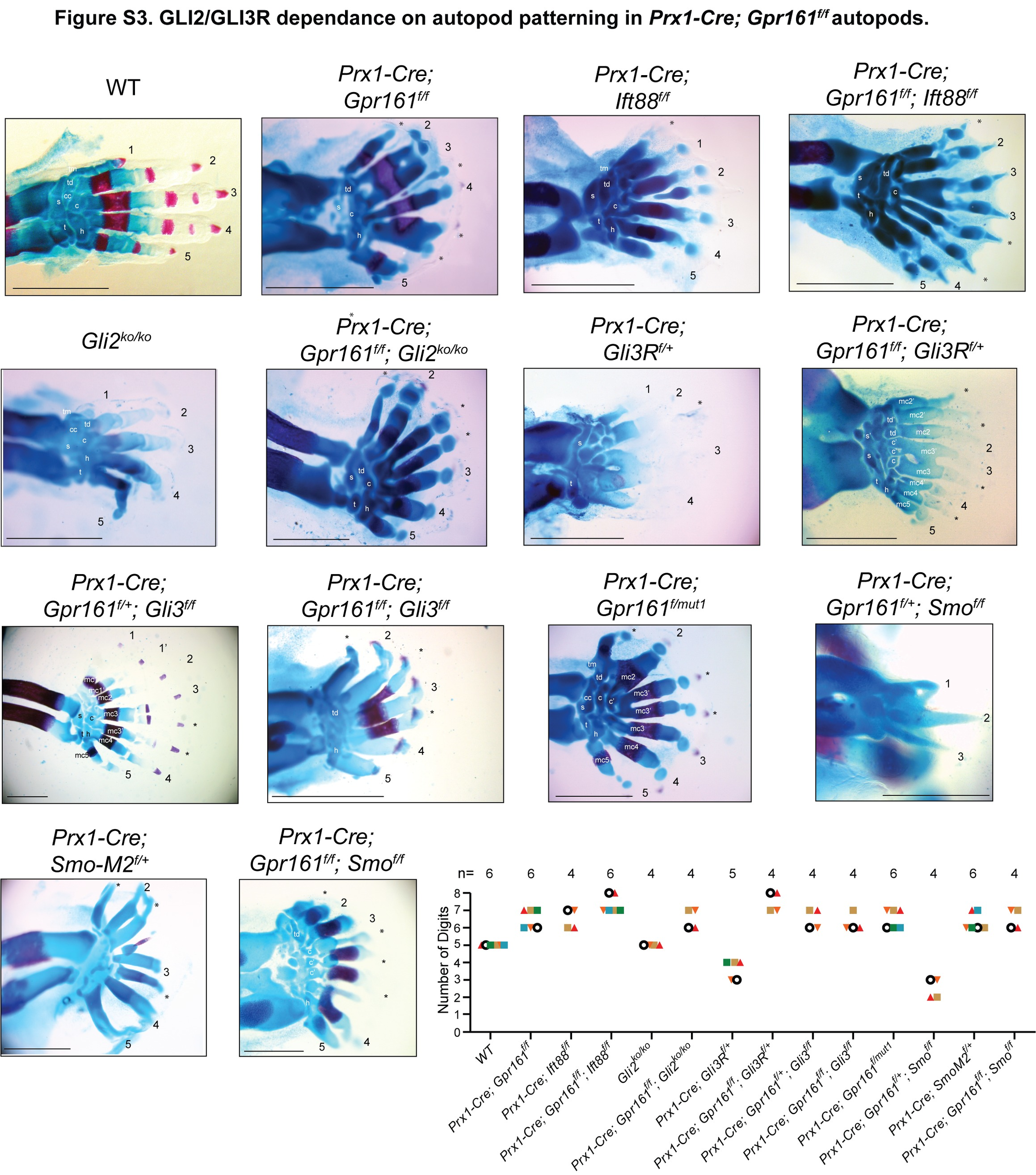

Supplement: S3 Fig — Alcian blue (unmineralized cartilage) and alizarin red (mineralized cartilage and bone) staining of E18.5 forelimb autopods. Duplicated or unassigned digits are marked by asterisks, whereas replicated carpals/metacarpals are hyphenated with likely identities. Quantification of whole digit numbers (without including duplicated phalanges or thin elongations) are shown below right. Each shape represents an individual autopod quantified and total number of autopods quantified (n) are shown on top of the graph. Abbreviations: c, Capitate; cc, Central carpal; h, Hamate; mc, Metacarpal; s, Scaphoid; td, Trapezoid; tm, Trapezium; t, Triquetral. Scale, 1 mm. (TIF) [file pgen.1011028.s003.tif]

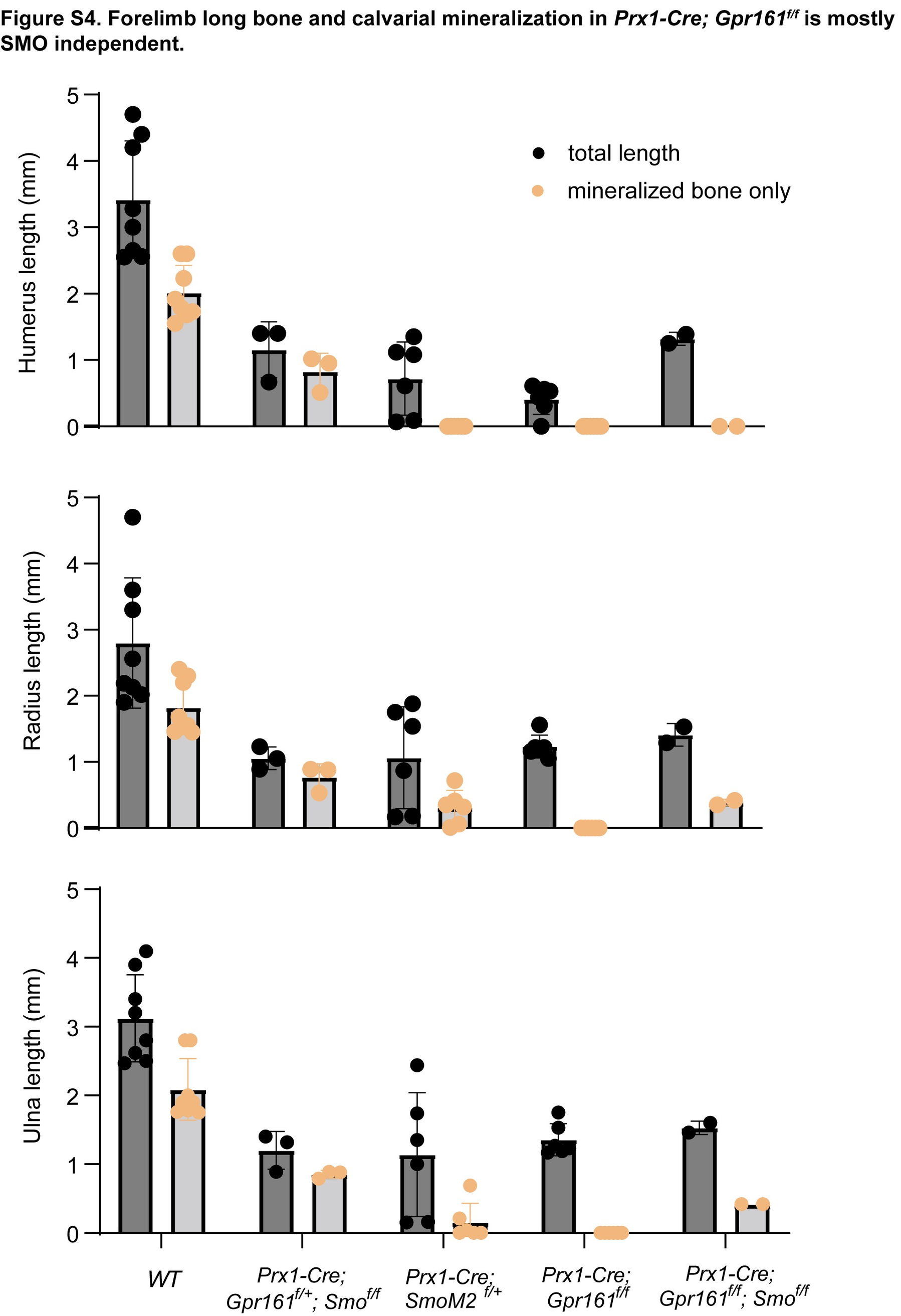

Supplement: S4 Fig — Quantification of forearm long bone primordia lengths in Fig 6 shown. Each data point represents individual bone primordia quantified. (TIF) [file pgen.1011028.s004.tif]

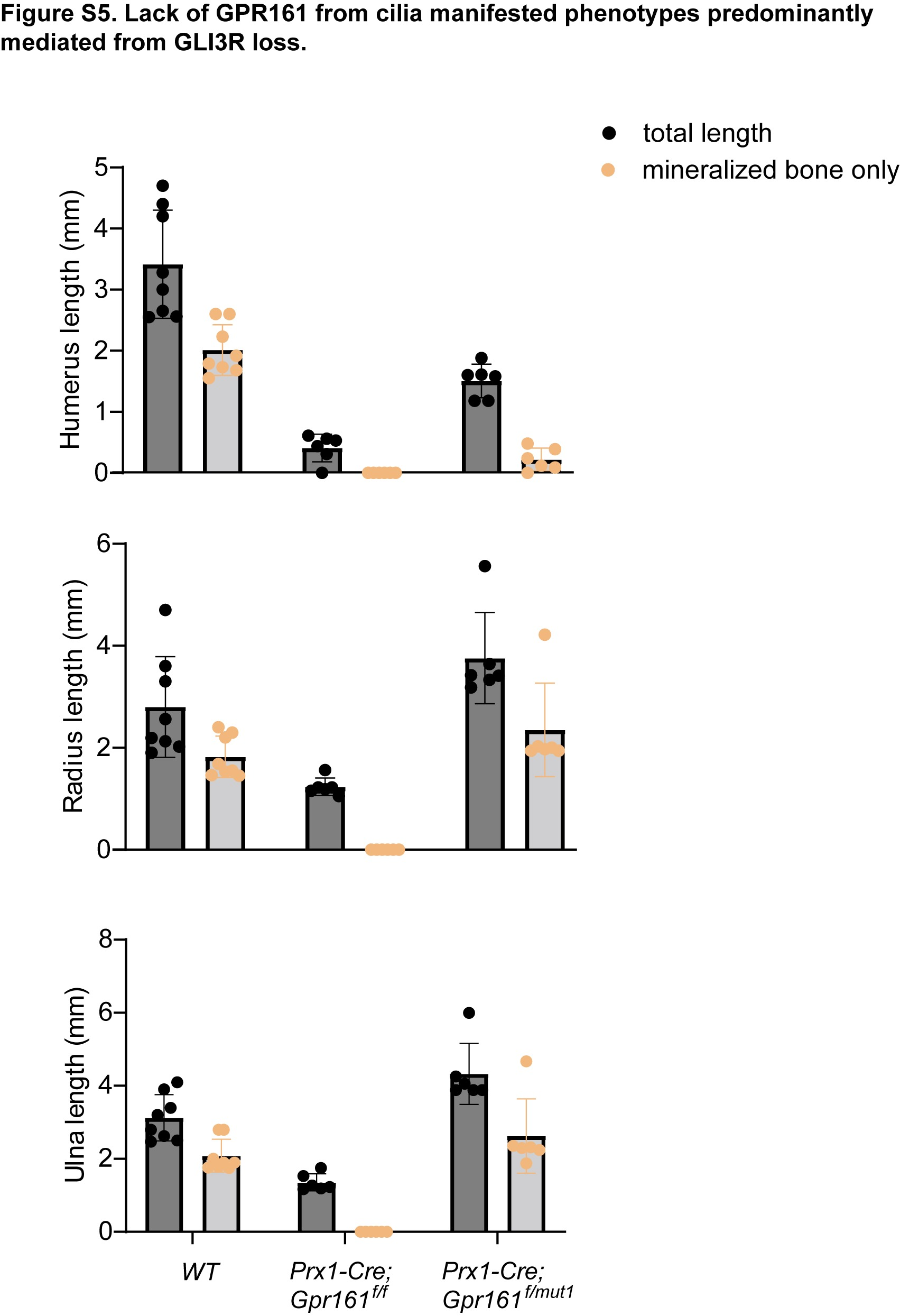

Supplement: S5 Fig — Quantification of forearm long bone primordia lengths in Fig 7 shown. Each data point represents individual bone primordia quantified. (TIF) [file pgen.1011028.s005.tif]
