# Supplementary material for: Context-dependent ciliary regulation of hedgehog pathway repression in tissue morphogenesis
Source: PLoS Genet. 2023 Nov 9;19(11):e1011028. doi: 10.1371/journal.pgen.1011028 (PMC10662714; doi:10.1371/journal.pgen.1011028)
Supplement: Attachment — Submitted filename: Revision PLOS Response_Hwang2.pdf [file pgen.1011028.s007.pdf]

We wholeheartedly thank the reviewers for their comments and constructive critique. We appreciate the very positive response from the editors and reviewers (for e. g., Reviewer 1: "provides a valuable framework", "data is of high technical quality and I am overall enthusiastic"; Reviewer 2: "a high-quality and thorough genetic analysis that is suitable for publication in PLOS Genetics"; and Reviewer 3: "work generally seems very sound and merits publication in PLOS Genetics").

We believe that we have now addressed most of the remaining concerns and revised the manuscript according to the suggestions. These revisions have resulted in the following changes:

- Textual edits and clarifications regarding homozygous *Gli3R* model generation (lines 468-473, lines 497-501, lines 141-142).
- Addition of quantification of NKX2.2 ventralization in Figs 2A", B" and 3A", B".
- Updated images for NKX2.2 ventralization in Figs 2 and 3.
- Updated numbers of animals from each genotype in all relevant Figs 2-7 and Fig S1.
- Updated images and annotation for showing lack of humerus mineralization in *Prx1-Cre; Gpr161*$^{f/f}$ in Figs 4-7.
- Updated *Gli2* ko image in Fig 4E.
- Updated quantification of autopods in Fig. S3.
- Other textual changes for (a) Quantification of phenotypes in Methods (lines 538-541), (b) Limitations of current mouse models (lines 240-242, lines 410-411), and (c) future directions (lines 435-437).

Revised text in the manuscript is shown by track changes and are also highlighted to show the requested revisions.

Reviewer #1: In this study, Hwang and colleagues examined the relative contributions of GLI-Activator (GLI-A) and GLI-Repressor (GLI-R) to determine how they regulate development in three different tissues. Using mutations that limit GLI function, they then examine the consequences of restoring GLI-R or GLI-A to the observed phenotypes of the neural tube, long bones and calvaria. This study is asking an important longstanding question about how Hedgehog signaling regulates different tissues in different ways. As aspects of both the Ankmy2 and Gpr161 phenotype characterizations are partially redundant with previous studies, the data is not all novel but there is real value in aggregating this into one focused study. The data is of high technical quality and I am overall enthusiastic about it although I do have some concerns with the study in its present form. First, while the neural tube provides a well-defined system for examining GLI readouts, interpreting the calvaria and long bone phenotypes is trickier because bone mineralization is a rather vague phenotype and it is unclear when GLI proteins regulate these tissues and what specific cell types are impacted. In addition, I am concerned about the reliance on GLI3-R/+ heterozygous embryos instead of GLI-R/R homozygous compound crosses that would provide more definitive information about the role of GLI repression. Despite these concerns and a few additional minor points all outlined below, this study provides a valuable framework for understanding how GLI-A and GLI-R interact with distinct mechanisms in different tissues.

Thanks for the positive comments.

Major concerns:

1. The study relies on GLI3-R/+ heterozygous embryos instead of GLI-R/R homozygous embryos for interpreting phenotypes caused by GLI repression. But as described by the Wang lab, heterozygous GL3-R/+ embryos do not fully copy the Shh-/- embryos like the homozygous alleles, indicating that both copies of GLI3-R are required for full GLI repression. While this does not need to be examined for all mutants/condition, it is important to determine if the dorsal NKX2.2 in the

Ankmy2-/-;GLI3R/+ neural tube goes away completely in Ankmy2-/-;GLI3R/R as predicted by the model.

Please note that as originally mentioned in the paper describing the conditional *Gli3*$^{\Delta701}$ (Gli3$^{\Delta701C}$) knock-in allele [1], heterozygotes of the ubiquitously recombined *Gli3*$^{\Delta701C}$ allele are embryonic lethal and are therefore not viable and suitable for breeding. The authors were unable to generate viable *Gli3*$^{+/\Delta701C}$*; Actin-Cre* animals by crossing *Gli3*$^{\Delta701C/\Delta701C}$ or *Gli3*$^{+/\Delta701C}$ with *Actin-Cre* for ubiquitous *Cre* expression (at least 70 mice were genotyped) [1]. Part of the confusion in interpreting the results might arise from the depiction of non-embryonic lethal *Prx1-Cre; Gli3*$^{\Delta701C/+}$ as "*Gli3*$^{\Delta701/+}$ " and *Prx1-Cre; Gli3*$^{\Delta701C/\Delta701C}$ as "*Gli3*$^{\Delta701/\Delta701}$" in the text and in Figures 2-4 in the original paper [1]. The inability to generate viable heterozygotes of the ubiquitously recombined *Gli3*$^{\Delta701}$ also demonstrate that the *Gli3*$^{\Delta701}$ allele produces a much more potent form of GLI3 repressor than the widely used *Gli3*$^{\Delta699}$ allele yielding viable heterozygotes [2]. Incidentally, homozygotes of *Gli3*$^{\Delta699}$ allele are also perinatal lethal [2].

We were also unable to generate viable animals with ubiquitously recombined *Gli3*$^{\Delta701C}$ allele using *CAG-Cre*. Therefore, mice breeders homozygous for *Gli3*$^{\Delta701C}$ were crossed with *CAG-Cre* [3] or *Prx1-Cre* [4] along with *Ankmy2* knockout (ko), *Gpr161* ko or *Gpr161*^{f} alleles to generate *Gli3R* containing genotypes in *Ankmy2* ko, *Gpr161* ko or conditional ko background, respectively. We have updated these facts in lines 468-473 and updated genotyping information in lines 497-501. We also clarified the confusion regarding homozygous *Gli3*$^{\Delta701/\Delta701}$ embryos in the original paper [1] in the text in lines 141-142 by clearly stating them to be *Prx1-Cre* expressing embryos homozygous for the conditional *GLI3*$^{\Delta701}$ allele. One way to alleviate this limitation might be to use epiblast specific *Cre* recombination [5], way beyond the scope of the current paper. Because of the requirement of the current breeding format, arising from the fact that the heterozygotes of the ubiquitously recombined *Gli3*$^{\Delta701}$ allele are not viable, we are unable to generate *Ankmy2* ko; *Gli3*$^{\Delta701/\Delta701}$ embryos, as suggested by the reviewer, or *Gpr161* ko; *Gli3*$^{\Delta701/\Delta701}$ embryos.

One of the phenotypes that the reviewer mentioned regarding testing for rescue using homozygozed *Gli3R* alleles, was the NKX2.2 ventralization in the neural tube of *Ankmy2* ko embryos. We agree that homozygous alleles of *Gli3R* could likely result in more complete rescue of FOXA2/NKX2.2 ventralization. However, the apparent persistent ventralization of NKX2.2 in *Ankmy2* ko; *Gli3R/+* and *Gpr161* ko; *Gli3R/+* in Fig 2 and Fig 3, respectively, was because of high background when using the anti-NKX2.2 antibody. We apologize for the poor quality of the NKX2.2 images we had before. We have now improved these images in Fig 2A-B and Fig 3A. We also added quantification for the dorso-ventral extent of NKX2.2 distribution in both thoracic and lumbar regions in Fig 2A"/B" and Fig 3A"/B". Essentially, the quantification shows that NKX2.2 ventralization in both *Ankmy2* and *Gpr161* ko is almost completely suppressed in the lumbar level similar to wild-type but less so in the thoracic levels, as we had already demonstrated for FOXA2.

Because of technical limitations in generating the *Gli3R* homozygous alleles, and more importantly, as we already see complete suppression of NKX2.2 ventralization in the lumbar level by one copy of *Gli3R* in both *Ankmy2* and *Gpr161* ko, we respectfully argue that having homozygous alleles for Gli3R will likely not add much to the current narrative.

2. The lack of bone mineralization used as a phenotype for GLI-A and GLI-R requirements is problematic for two reasons. First, this is an indirect readout and it is unclear what is the affected cell type (osteoblast specification? chondrocyte morphology and maturation?). Second it is unclear when GLI signaling regulates bone development. Does this occur sequentially in the limb bud and then in the bone, a complicating factor in considering ratio sensing? Are the missing skull bones a consequence of direct or indirect Hedgehog signaling? And how are crest and nonneural crest derived tissues affected at the same time? These caveats should be acknowledged and discussed both within the results and the discussion.

Thanks for these important comments on the skeletal phenotypes. We previously showed a complete lack of mineralization in the forearm long bones of *Prx1-Cre; Gpr161$^{f/f}$* embryos [6]. The chondrocytes did not progress beyond the periarticular chondrocyte stage, were ciliated, and showed no IHH expression. We also demonstrated lack of calvarium intramembranous bone formation [6]. As the reviewer states, the exact cell type(s) directly affected from *Gpr161* loss in manifestation of these phenotypes are not currently known. In collaboration with Dr. Courtney Karner, UT Southwestern, we have performed calvarial mesenchymal cell differentiation assays, and we see a reduction in osteogenesis in cultured primary cells in *Gpr161* ko. However, we don't see osteoblast-specific *Sp7-Cre; Gpr161$^{f/f}$* mice to manifest the skeletal phenotypes as in *Prx1-Cre; Gpr161$^{f/f}$* mice, suggesting that *Prx1/Dermo* positive osteo-chondro progenitors are most likely affected from Gpr161 loss. In line with these results, GPR161 localizes to the cilia of mesenchymal stem cell lines [7]. We are currently planning to use *Dermo/Col2a/Runx2-Cre* to determine the specific cell type affected in these phenotypes. These results are beyond the scope of the current paper. We have now highlighted these caveats in results (lines 240-42). However, both the skeletal phenotypes were rescued from concomitant lack of cilia. Thus, these phenotypes are important for dissecting modes of cilia-mediated HH repression.

In line with the comment whether crest and nonneural crest derived tissues were affected at the same time, the cranial vault (calvarium) and facial bones are known to arise directly from deep layers of the dermis via intramembranous ossification [8]. As the reviewer elutes to, in the mouse, the calvarial bones posterior to the coronal suture (posterior skull), except a part of the interparietal bones, are derived from cranial mesoderm, whereas frontal bone and facial bones are derived from the cranial neural crest cells [9]. We previously showed *Prx1-Cre* expression in

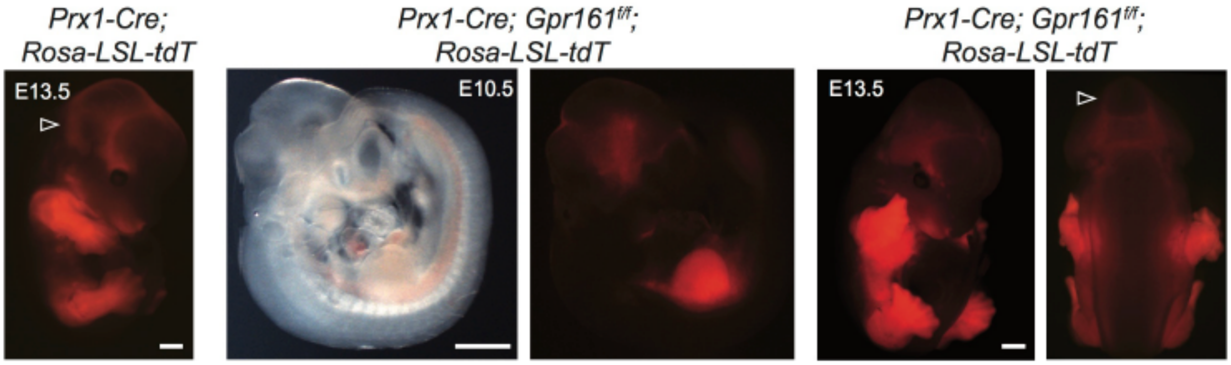

Fig R1. *Prx1-cre; Rosa26-loxP-STOP-loxP-tdTomato* (*Rosa-LSL-tdT*) embryos depict tdTomato positive cranial mesenchyme (arrowhead). All lateral views, except right panel (*en face*). Scale, 1 mm. (From our previous paper [Hwang et al, Dev 2018] [6] Fig S3A).

cranial mesenchyme (Fig. S3A) [4, 10], overlapping both crest and nonneural crest derived regions (Fig R1 adapted from [6]). Coincidentally, there was a complete lack of posterior skull and posterior frontal mineralization in *Prx1-Cre*; *Gpr161$^{f/f}$* (Fig. 5B) [6], whereas anterior frontal, facial and mesoderm-derived endochondral bones in base of skull (basioccipital/basisphenoid) remained unaffected [6].

Minor issues:
3. Figures 2 and 3. In contrast to other markers, it isn't easy to see the signal in all the NKX2.2 panels (both in Figs. 2 and 3) and this is critical for interpreting GLI-R readouts. Higher resolution images, perhaps without DAPI overlay might help resolve this.

We apologize for the poor quality of the NKX2.2 images in Fig 2 and Fig 3. This is because of high background when using this antibody. We have now improved these images in Fig 2A-B and Fig 3A and added quantification for the dorso-ventral extent of NKX2.2 distribution in Figs 2 and 3. Essentially, the quantification shows that NKX2.2 ventralization in *Ankmy2* and *Gpr161* ko is almost completely suppressed in the lumbar level similar to wild-type but less so in the thoracic levels, as we already demonstrated for FOXA2.

4. Figure S1. Does not indicate the number of embryos examined per genotype. This should be added to the legend.

We have added number of embryos examined per genotype in all relevant Figs (2-7) and Supp Figs (S1, 3).

5. Typo (stray |) on line 197

Corrected, thanks.

6. Figure 4A,B and line 252. It is stated that the length of the humerus is reduced in Prx1-Cre;Gpr161f/f forelimbs but it isn't possible to clearly see the humerus in the image provided and it isn't clear to me from looking at 4B that it is even present. Could this be replaced with a clearer image?

We previously showed that humerus is not mineralized in *Prx1-Cre; Gpr161*$^{f/f}$ embryos (Fig R2, adapted from [6]). We have now annotated and/or updated the images to show the unmineralized humerus in Fig 4B, 6F-H and 7C.

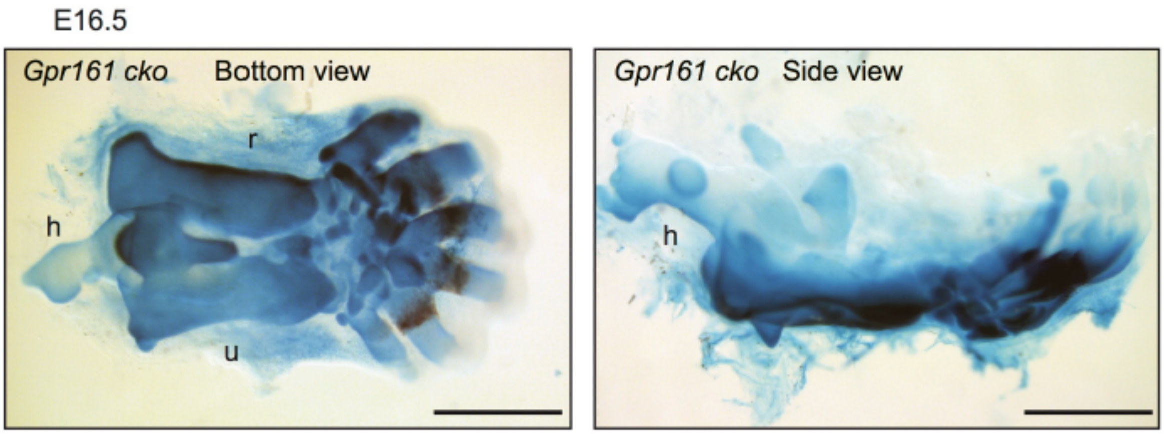

Fig R2. *Prx1-Cre; Gpr161*$^{f/f}$ embryos shows unmineralized long bones (From our previous paper: Fig S3B [Hwang et al, Dev 2018] [6]). h, humerus; r, radius; u, ulna.

7. Figure 4E. The humerus does not see completely mineralized as indicated on the schematic lying directly below, which seems to be a mistake based on their stated model.

Apologies for the confusion. We have improved the image in Fig 4E to show that the humerus in *Gli2* ko is mineralized similar to wild-type (also quantified in Fig 4K).

8. Figure S3. State the N's for each specific cross in the figure legend and also to what degree the phenotypes are penetrant. Since there is highly likely to be variation, it would be nice to quantify each of these mutant conditions to show their penetrance.

Thanks for the suggestion. We have added quantification for number of digits in Fig S3 to show the penetrance of the phenotypes.

9. Figure 5 legend. Please add N's for each specific genotype.

Thanks for the suggestion. We have added N's for specific genotypes in all relevant Figs (2-7) and Supp Figs (S1, 3).

Reviewer #2: This manuscript from Mukhopadhyay and colleagues focuses on the GLI2 and GLI3 proteins, which serve as the major transcriptional effectors of the Hedgehog (Hh) pathway. GLI2 and GLI3 are bifunctional proteins that can exist in activator or repressor forms; while cilia are needed for both GLI repressor and activator formation, the degree to which ciliary Hh signaling (and cilium-related phenotypes) rely on repressors, activators, or both remains incompletely understood.

To address these questions, the authors employ genetic and embryological approaches to investigate the roles of GLI2 and GLI3 during morphogenetic Hh signaling in two well-established experimental models -- neural tube and skeletal development. They took advantage of two genetic manipulations that derepress ciliary Hh signaling to different extents: 1) loss of Ankmy2, which serves to traffic adenylyl cyclases to cilia; 2) loss or ciliary delocalization of Gpr161, a constitutively active GPCR that helps to set baseline PKA activity in cilia. To study the role of GLI activators and repressors, the authors combined the above genetic manipulations with either: 1) enforced expression of GLI3 repressor (GLI3R); 2) loss of Gli2, the major transcriptional activator of the Hh pathway. The authors go on to conduct a careful and comprehensive epistasis analysis, including appropriate controls and quantifications.

The key finding is that during neural tube and skeletal development, some Gpr161 or Ankmy2 phenotypes are rescued by either GLI3R expression or Gli2 knockout, whereas others can only be rescued by one or the other. Based on this finding, the authors articulate a model, building on the one they proposed in their 2ti21 eLife paper, that three distinct modes of GLI target gene regulation (ratio sensing, GLI3R threshold, and GLI2A threshold) carry out the transcriptional effects of Hh signaling.

Overall this is a high-quality and thorough genetic analysis that is suitable for publication in PLOS Genetics, pending the following minor revisions to the text and figures:

1. The authors assume that GLI2 functions primarily as an activator in their experiments. Is it possible that GLI2 repressor plays any roles? A limitation of Gli2 knockout is that it removes both GLI2R and GLI2A. This is a reasonable assumption because GLI2 undergoes far less proteolytic processing to a repressor than does GLI3. Nevertheless, given that the authors made a point of emphasizing how a Gli2 or Gli3 knockout cannot distinguish between activator and repressor roles (p. 4-5), and given that the authors don't have a way to explicitly test GLI2R like they do with the GLI3(delta701) allele, it's worth acknowledging this possibility as a caveat of the GLI2 knockout studies.

Thanks for the overall positive comments and the current suggestion. We already mentioned in the Discussion (lines 408-410) that lack of cilia in the face causes widening of the face by increasing distance between frontonasal processes that phenocopies *Gli2; Gli3* double ko (but not single ko of *Gli2/3*) or *Gpr161* conditional ko [11, 12]. *Gli3R* expression rescues this phenotype, suggesting predominant role of both GLI-2R and GLI-3R [12]. In the absence of a specific allele generating GLI-2R, we are unable to explicitly test the role of GLI-2R. We now mention this caveat in the Discussion (lines 410-411).

2. It is interesting that the Ankmy2 exencephaly phenotype is not overcome by either GLI3R expression or Gli2 knockout, and therefore doesn't appear to fall into one of the 3 modes of GLI regulation in the authors' model. Can the authors offer any potential explanation for this result?

Thanks for highlighting this interesting point. We do see rescue of exencephaly in *Gpr161* ko by expressing *Gli3R* but not in the background of *Gli2* ko (unpublished). However, considering the range of phenotypes we have already discussed, we respectfully suggest that cranial neural tube defects and forebrain development is beyond the scope of the current paper. In the same line, the lack of rescue of exencephaly by *Gli3R* in *Ankmy2* ko is likely from severity of the phenotype and both alleles of *Gli3R* might be required for restoring the phenotype. As we mentioned in response to Reviewer 1, we are unable to do this experiment currently due to technical limitations.

3. I am confused about blue color scheme in the cartoons for the calvarium studies (i.e., Fig 5D) and what it's supposed to represent. Please clarify this in the figure legend.

We apologize for the confusion. We have retained the color scheme for the parietal bone instead of resorting to a blue color scheme.

4. There are a large # of mouse strains and phenotypes to keep track of in this study. To improve readability and comprehension, I would suggest the authors summarize their findings in a supplementary table.

Thanks for the suggestion. We have shown relevant quantification of the data in the figures and summarized the key points regarding the complex phenotypes in associated figure schematics in Figs 2-7. Finally, a summary of the study scheme with strains used and phenotypes are presented in the Fig 1 and Fig 8 schematics, respectively. Therefore, we respectfully suggest to not have another supplementary table enumerating similar points.

Reviewer #3: Hwang et al. presents a detailed genetic study examining the relative contributions of GliA and GliR in the Hh-dependent pa4erning of several tissues: the neural tube, limbs, and calvarium. The manuscript is very well written and reader-friendly. While the topic of tissue patterning by differential regulation of GliA/GliR rations has certainly been examined previously, the genetic epistasis experiments presented here have generated new insights and led to a fairly simple model to explain how tissues respond differently to either different thresholds or different rations of GliA and GliR. This work generally seems very sound and merits publication in PLoS Genetics.

Thanks for the positive comments.

I have only fairly minor points:

I appreciate that the authors quantified their results both for neural patterning and bone mineralization, but some methodological description of how these measurements were taken would be helpful. I don't see this in the Methods section.

Thanks for the suggestion. We have now mentioned the description of how these measurements were conducted in a new section at the end of the Methods (lines 538-541).

I was intrigued by the fact that while most of the tissues examined use the ratio sensing mode in their Hh response, some cell types use the threshold modes. I wonder if the authors think their model could be used to make predictions about the mode of response in other cell types, and how this might relate to cilia. For example, in cell types like the fibro-adipogenic progenitors described in Kopinke et al.(2017), where the presence of a cilium is suppressing Hh signaling.

We apologize for the oversight. We have now included in the Discussion that future studies are needed to determine if these modes of regulation are relevant during hedgehog pathway mediated tissue regeneration, such as in muscle-resident fibro/adipogenic progenitors [13] (lines 435-437) .

References

1. Cao T, Wang C, Yang M, Wu C, Wang B. Mouse limbs expressing only the Gli3 repressor resemble those of Sonic hedgehog mutants. Dev Biol. 2013;379(2):221-8. doi: 10.1016/j.ydbio.2013.04.025. PubMed PMID: 23644062; PubMed Central PMCID: PMCPMC3707282.

2. Bose J, Grotewold L, Ruther U. Pallister-Hall syndrome phenotype in mice mutant for Gli3. Hum Mol Genet. 2002;11(9):1129-35. Epub 2002/04/30. doi: 10.1093/hmg/11.9.1129. PubMed PMID: 11978771.

3. Sakai K, Miyazaki J. A transgenic mouse line that retains Cre recombinase activity in mature oocytes irrespective of the cre transgene transmission. Biochem Biophys Res Commun. 1997;237(2):318-24. Epub 1997/08/18. doi: 10.1006/bbrc.1997.7111. PubMed PMID: 9268708.

4. Logan M, Martin JF, Nagy A, Lobe C, Olson EN, Tabin CJ. Expression of Cre Recombinase in the developing mouse limb bud driven by a Prxl enhancer. Genesis. 2002;33(2):77-80. doi: 10.1002/gene.10092. PubMed PMID: 12112875.

5. Perez-Garcia V, Fineberg E, Wilson R, Murray A, Mazzeo CI, Tudor C, et al. Placentation defects are highly prevalent in embryonic lethal mouse mutants. Nature. 2018;555(7697):463-8. Epub 20180314. doi: 10.1038/nature26002. PubMed PMID: 29539633; PubMed Central PMCID: PMCPMC5866719.

6. Hwang SH, White KA, Somatilaka BN, Shelton JM, Richardson JA, Mukhopadhyay S. The G protein-coupled receptor Gpr161 regulates forelimb formation, limb patterning and skeletal morphogenesis in a primary cilium-dependent manner. Development. 2018;145(1). Epub 2017/12/10. doi: 10.1242/dev.154054. PubMed PMID: 29222391; PubMed Central PMCID: PMCPMC5825871.

7. Johnson GP, Fair S, Hoey DA. Primary cilium-mediated MSC mechanotransduction is dependent on Gpr161 regulation of hedgehog signalling. Bone. 2021;145:115846. Epub 20210113. doi: 10.1016/j.bone.2021.115846. PubMed PMID: 33450431.

8. Abzhanov A, Rodda SJ, McMahon AP, Tabin CJ. Regulation of skeletogenic differentiation in cranial dermal bone. Development. 2007;134(17):3133-44. doi: 10.1242/dev.002709. PubMed PMID: 17670790.

9. Chai Y, Maxson RE, Jr. Recent advances in craniofacial morphogenesis. Dev Dyn. 2006;235(9):2353-75. doi: 10.1002/dvdy.20833. PubMed PMID: 16680722.

10. Goodnough LH, Chang AT, Treloar C, Yang J, Scacheri PC, Atit RP. Twist1 mediates repression of chondrogenesis by beta-catenin to promote cranial bone progenitor specification. Development. 2012;139(23):4428-38. doi: 10.1242/dev.081679. PubMed PMID: 23095887; PubMed Central PMCID: PMCPMC3509735.

11. Hwang SH, Somatilaka BN, White K, Mukhopadhyay S. Ciliary and extraciliary Gpr161 pools repress hedgehog signaling in a tissue-specific manner. Elife. 2021;10. Epub 2021/08/05. doi: 10.7554/eLife.67121. PubMed PMID: 34346313; PubMed Central PMCID: PMCPMC8378848.

12. Chang CF, Chang YT, Millington G, Brugmann SA. Craniofacial Ciliopathies Reveal Specific Requirements for GLI Proteins during Development of the Facial Midline. PLoS Genet. 2016;12(11):e1006351. Epub 2016/11/02. doi: 10.1371/journal.pgen.1006351. PubMed PMID: 27802276; PubMed Central PMCID: PMCPMC5089743.

13. Kopinke D, Roberson EC, Reiter JF. Ciliary Hedgehog Signaling Restricts Injury-Induced Adipogenesis. Cell. 2017;170(2):340-51 e12. doi: 10.1016/j.cell.2017.06.035. PubMed PMID: 28709001; PubMed Central PMCID: PMCPMC5617351.
